# Supercapacitor performance of porous nickel cobaltite nanosheets

**DOI:** 10.1038/s41598-020-75946-1

**Published:** 2020-11-03

**Authors:** Xin Chen, Rui Xie, Hui Li, F. Jaber, F. Musharavati, E. Zalnezhad, S. Bae, K. S. Hui, K. N. Hui

**Affiliations:** 1 Department of Mechanical Convergence Engineering, Hanyang University, 222 Wangsimni-ro, Seongdong-gu, Seoul, 04763 South Korea; 2 Department of Electronic Information, Shandong Xiandai University, Jinan, 250000 People’s Republic of China; 3 Department of Chemical Engineering, Hanyang University, 222 Wangsimni-ro, Seongdong-gu, Seoul, 04763 South Korea; 4 Department of Biomedical Engineering, Ajman University, 2758, Ajman, UAE; 5 Department of Mechanical and Industrial Engineering, College of Engineering, Qatar University, 2713, Doha, Qatar; 6 Department of Chemical and Biomedical Engineering, University of Texas At San Antonio, San Antonio, TX USA; 7 Department of Architectural Engineering, Hanyang University, Seoul, 04763 Korea; 8 School of Engineering, University of East Anglia, Norwich, NR4 7TJ UK; 9 Joint Key Laboratory of the Ministry of Education, Institute of Applied Physics and Materials Engineering, University of Macau, Avenida da Universidade, Taipa, Macau SAR, 999078 China

**Keywords:** Energy storage, Nanoscale materials

## Abstract

In this work, nickel cobaltite (NiCo_2_O_4_) nanosheets with a porous structure were fabricated on nickel foam as a working electrode for supercapacitor applications. The nanosheets were fabricated by electrochemical deposition of nickel–cobalt hydroxide on the nickel foam substrate at ambient temperature in a three-electrode cell followed by annealing at 300 °C to transform the coating into a porous NiCo_2_O_4_ nanosheet. Field emission scanning electron microscopy and transmission electron microscopy revealed a three-dimensional mesoporous structure, which facilitates ion transport and electronic conduction for fast redox reactions. For one cycle, the NiCo_2_O_4_ electrodeposited nickel foam has a high specific capacitance (1734.9 F g^−1^) at a current density (CD) of 2 A g^−1^. The electrode capacitance decreased by only approximately 12.7% after 3500 cycles at a CD of 30 A g^−1^. Moreover, a solid-state asymmetric supercapacitor (ASC) was built utilising the NiCo_2_O_4_ nanosheets, carbon nanotubes, and a polyvinyl alcohol-potassium hydroxide gel as the anode, cathode, and solid-state electrolyte, respectively. The ASC displayed great electrochemical properties with a 42.25 W h kg^−1^ energy density at a power density of 298.79 W kg^−1^.

## Introduction

Due to rapidly increasing logical pollution, fossil fuel depletion, and the fast growth of the worldwide economy, it is vital to develop clean, sustainable, and efficient energy resources, along with new technologies for energy conversion and storage^1–5^. In recent years, ultracapacitors, or electrochemical supercapacitors (ESs), have generated substantial interest, owing mostly to their long lifecycles, high power density, and ability to bridge the power/energy gap in conventional batteries/fuel cells and dielectric capacitors because of their high energy storage capacity and high power output^6,7^.

Lately, many researches have concentrated on binary metal oxides. Their outstanding specific capacity and great electrical conductivity are better than single component oxides due to their attainable oxidation states for multiple redox reaction^8^. Furthermore, binary metal oxides have many other benefits, such as relative abundance, low cost, and environmental friendliness^9^. In recent years, researches have shown that binary metal oxides, such as nickel cobaltite (NiCo_2_O_4_), ZnCo_2_O_4,_ and Zn_2_SnO_4_ are favourable materials that show improved electrochemical performance. Moreover, they are scalable replacements owning to their ample surface active sites, high electrical conductivity, strong permeability, and attainable oxidation states. Thus, many works have been performed to synthesize dissimilar bimetallic oxide nanomaterials for supercapacitor applications with great rate capabilities. The binary metal oxide NiCo_2_O_4_ was recently studied for use as an electrode owing to its electrochemical activity, stability, and higher electronic conductivity compared to single metal oxides^10–13^. NiCo_2_O_4_ has a structure similar to Co_3_O_4_ (spinel structure), and both the Co and Ni ion have a mixed oxidation state^14,15^. The electrical conductivity of NiCo_2_O_4_ is more than twice that of the Co or Ni oxide alone because the replacement of Co with Ni brings additional electrons into the 3d orbital, which subtly changes the density of electrons in the crystal structure. The NiCo_2_O_4_ working potential window is often very slim, near 0–0.5 V, in comparison with alkaline solutions (Ag/AgCl)^16–19^. It has been proved that the introduction of graphene can effectively enhance the total capacitance and stability, primarily because graphene can withstand the basic structures of polyaniline and evade mechanical deformation in the redox process^20^. Therefore, ternary composites with carbon nanomaterials, using transition metal oxides and polymers, have been studied and showed an enhancement in the electrochemical performance that open a new fabrication pathway for next generation high-performance electrochemical electrodes^21^. Ternary CuCo_2_S_4_ is well known as an electrode material for electrochemical capacitors because of its synergistic effects, high conductivity, and low cost. The supercapacitive performance of CuCo_2_S_4_ electrodes for electrochemical capacitors was investigated by Xu et al. In their study, thin layers of CuCo_2_S_4_ were deposited on conductive substrates, and the results showed better properties than single crystal CuCo_2_S_4_^22^.

Electrodeposition has been utilised for different applications, such as microelectronics and energy conversion. Because of the need for enhanced performance and device miniaturization, nuanced control of the growth process is needed; electrochemical deposition is a technique that can fulfil those requirements^23^.

Chen et al. successfully deposited Ni–Co–S nanosheet arrays on carbon fibres through one-step electrodeposition of the ternary sulfides to provide an effective and facile approach for large scale applications, which was a significant advantage compared to other multistep synthesis techniques. They found that the Ni–Co–S–4 interconnected nanosheet exhibits the best electrochemical performance as a supercapacitor electrode^24^.

**Figure 1:**
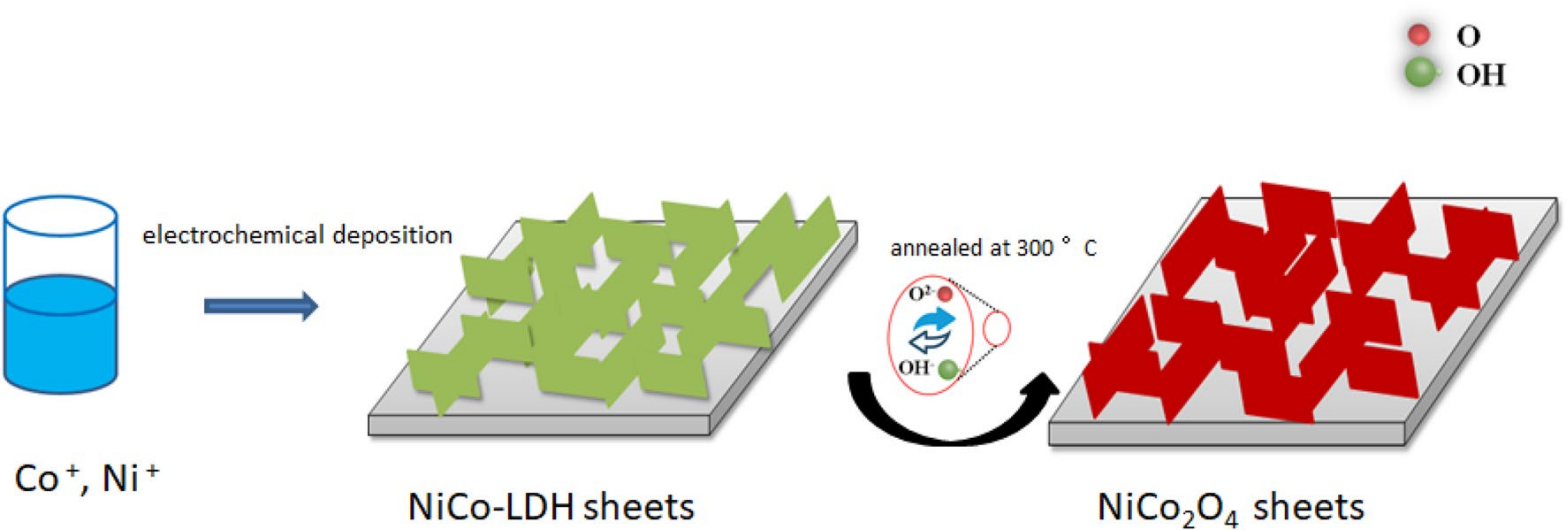
Schematic illustration of porous NiCo_2_O_4_ sheets synthesis.

One effective method of enlarging the potential window of the NiCo_2_O_4_ electrode to attain a high energy density is the utilisation of asymmetric devices^16,25–27^. For example, Yedluri et al. fabricated chain-like NiCo_2_O_4_/NiCo_2_O_4_ nanofile arrays using a facile hydrothermal and thermal decomposition approach and reported a specific capacitance of 2312 F g^−1^ at a current density (CD) of 2 mA cm^−2^^28^. They also studied the electrochemical performance of NiCo_2_O_4_@NiCo_2_O_4_ composite nanoplates and NiCo_2_O_4_ nanoplates decorated with NiMoO_4_ honeycombs for high performance supercapacitor applications^29,30^. Herein, we developed a solid asymmetric supercapacitor with a pre-synthesised NiCo_2_O_4_ mesoporous electrode without binders, where a nickel foam (with high conductivity) was selected as a current collector, and the NiCo_2_O_4_ provided a large surface area along with a unique mesoporous nanostructure. Our sample delivered a specific capacitance of 1734.9 F g^−1^ at a 2 A g^−1^ CD with a retention rate of 87.3% at a 30 A g^−1^ CD for 3500 cycles. Furthermore, the application of a NiCo_2_O_4_-based binder-free electrode to a solid asymmetric supercapacitor resulted in a 42.25 W h kg^−1^ energy density at a power density of 298.79 W kg^−1^ (Fig. 1).

## Results and discussion

**Figure 2:**
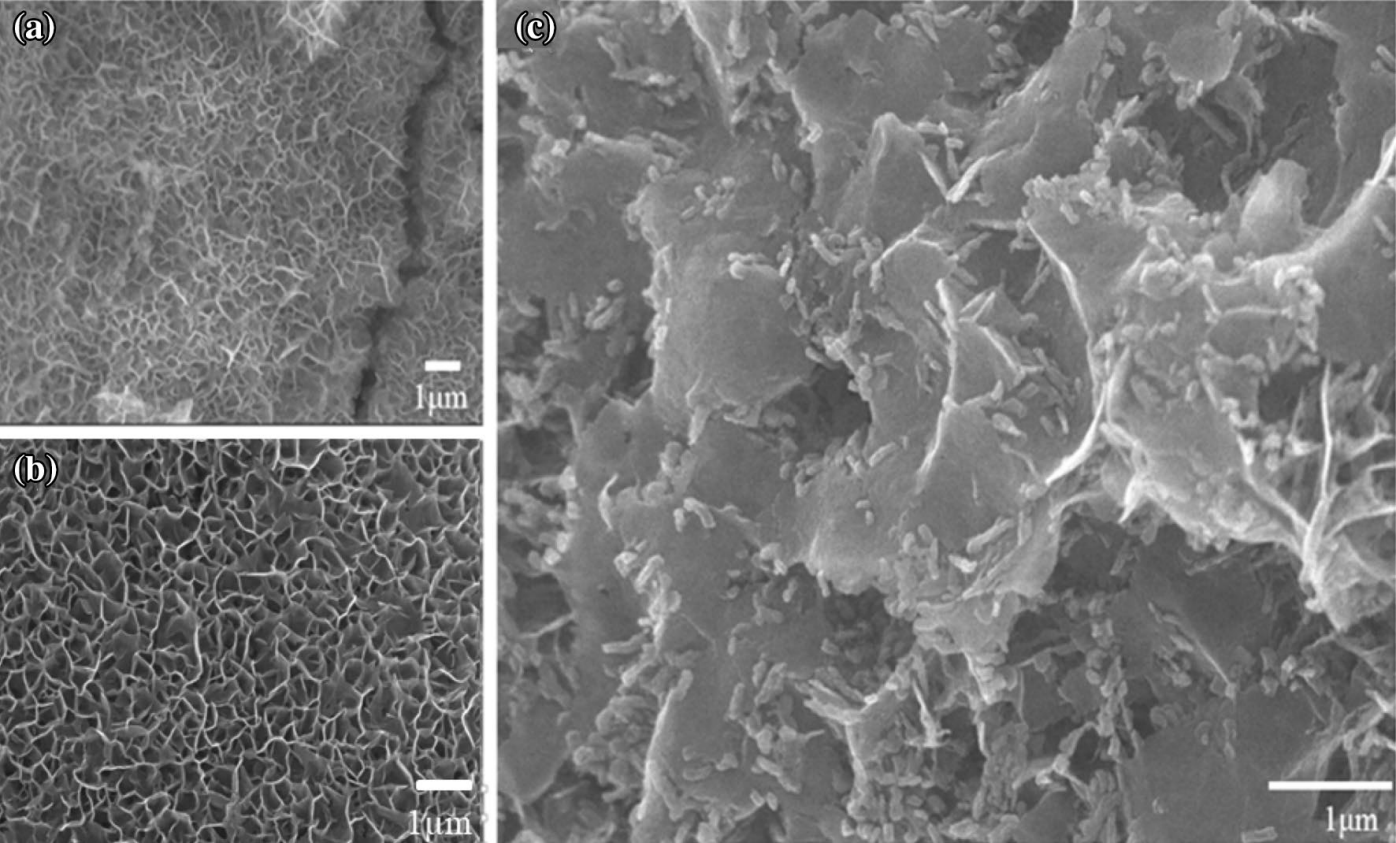
FESEM images of (**a**) Ni foam covered by the bimetallic (Ni, Co) hydroxide precursor, and (**b**, **c**) the derived NiCo_2_O_4__1 interconnected sheets.

Figure 2 depicts SEM images of NiCo-LDH and NiCo_2_O_4_. The images show that a smooth, uniform array of NiCo_2_O_4_ nanosheets is grown on the nickel foam surface, and the nanosheets are interlaced to form a mesoporous structure. Figure 2 (a) shows that the NiCo-LDH possesses an interconnected nanosheet microstructure. Figure 2(b, c) shows SEM images of NiCo_2_O_4_. These nanosheets, which are several hundred nanometers in size, have a porous structure that is intercrossed, which contains electroactive surface sites and plentiful vacancies^31–33^.

**Figure 3:**
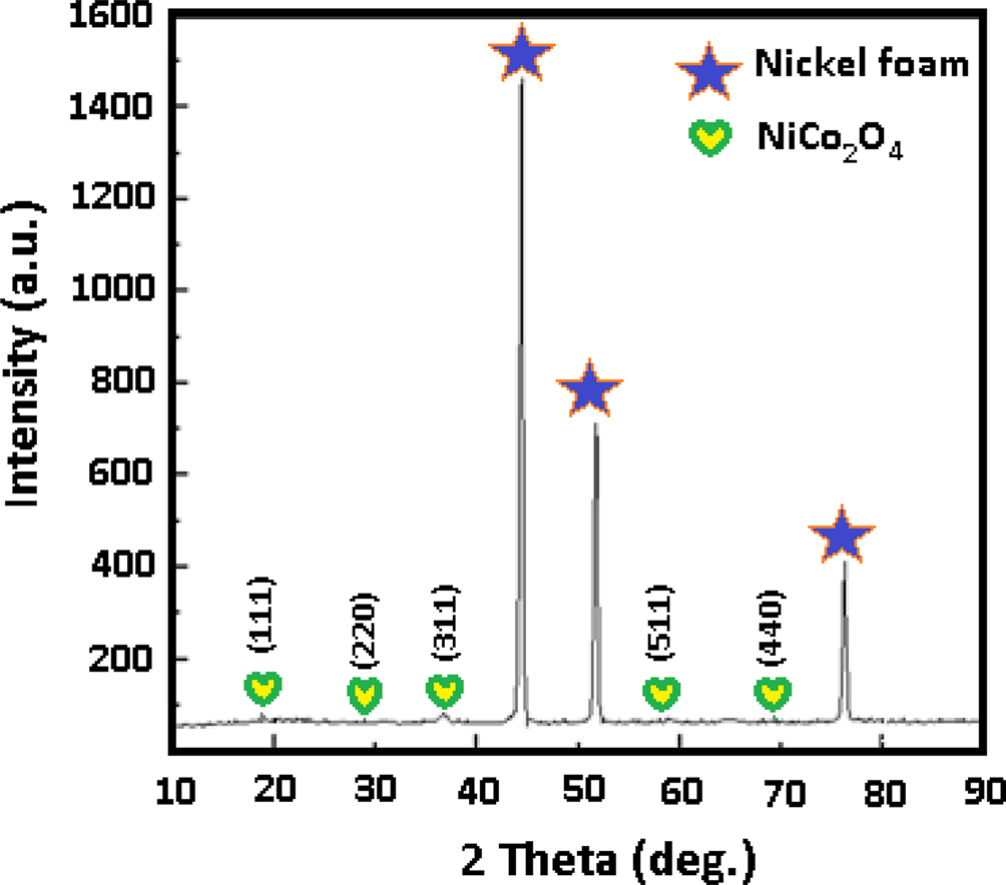
XRD pattern of NiCo_2_O_4_.

The products were further examined by X-ray diffraction (XRD) analysis. Figure 3 depicts the XRD pattern of the NiCo_2_O_4_ nanosheets deposited on the Ni foam. The peaks (from the (111), (200), and (220) planes, respectively) at 44.7°, 52.1°, and 76.5°, denoted by asterisks, are created from the nickel foam. The peaks at 18.9°, 36.6°, 59.1°, and 64.9° can be clearly observed and are well indexed to the (111), (311), (511), and (440) planes, respectively, belonging to NiCo_2_O_4_ (JCPDS Card No. 20-0781)^34,35^.

**Figure 4:**
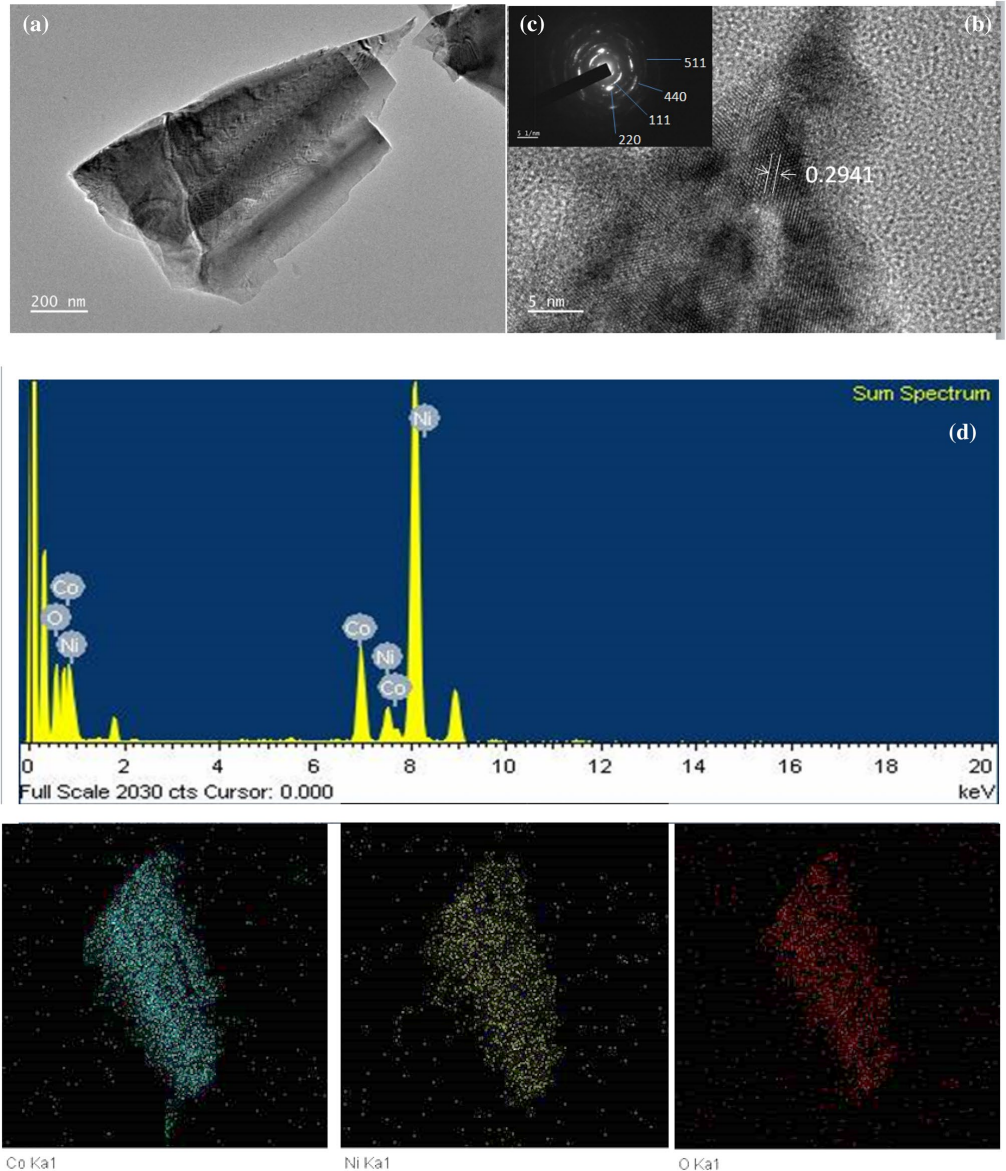
(**a**) TEM image and (**b**) HRTEM image of an individual NiCo_2_O_4_ nanosheet, along with (**c**) the SAED pattern (inset), and (**d**) the corresponding EDS mapping results.

Transmission electron microscopy (TEM) measurements were performed to further investigate the structure of the synthesised NiCo_2_O_4_ nanosheets, as shown in Fig. 4. Figure 4(a, b) shows a NiCo_2_O_4_ nanosheet with a folding, silk-like morphology and transparent features, indicating its interconnected nature. Due to the significant difference between the lateral size and thickness, bending and crumpling are clearly observed. The spacing between adjacent fringes is ~ 0.29 nm, which is close to the theoretical interplane spacing of spinel NiCo_2_O_4_ (311) planes. Thus, the interconnected nanosheets are composed of 1–3 layers of NiCo_2_O_4_ atomic sheets. The selected area electron diffraction (SAED) pattern (Fig. 4 (c)) presents distinct diffraction rings, indicating polycrystalline characteristics. Furthermore, several interparticle mesopores, with sizes ranging from 1 to 3 nm, in these interconnected nanosheets can be evidently seen (Fig. 4 (a,b)). It is believed that the mesoporous structures in nanosheets are imperative in facilitating the electrolytes’ mass transport within the electrodes for double-layer charging/discharging and quick redox reactions^8^. The porous structure also significantly increases the contact area of electrolyte/electrode, and consequently enhances the electrochemical performance^9–15^. To show the advantages of this architecture, the interconnected mesoporous NiCo_2_O_4_ nanosheets with hybrid structure fabricated on Ni foam was directly applied as an electrode for a supercapacitor. Figure 4(d) show the energy dispersive X-ray spectroscopy (EDS) analysis of the NiCo_2_O_4_ sample; Co, Ni, and O are detected. This result confirms the chemical composition of the NiCo_2_O_4_ structure.

**Figure 5:**
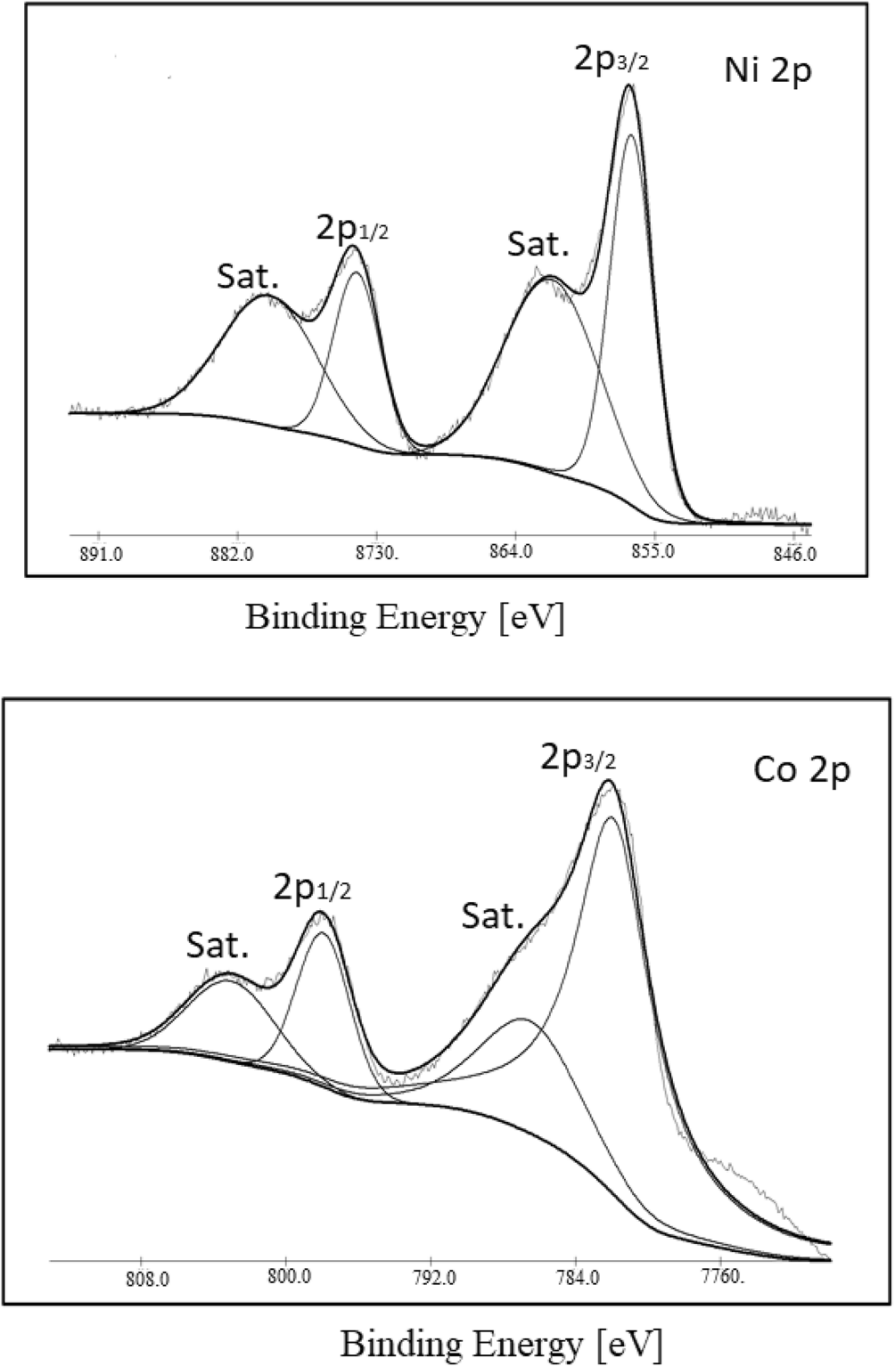
High-resolution XPS spectra of Ni 2p and Co 2p.

X-ray photoelectron spectroscopy (XPS) tests, along with the corresponding fitting results are shown in Fig. 5. Gaussian fitting method was used to best fit the Ni 2p and Co 2p (with two spin–orbit doublets for each), characteristic of Ni^2+^ and Ni^3+^ and Co^2+^ and Co^3+^, respectively and two shake-up satellite (indicated as “Sat.”) for both Ni 2p and Co 2. These data show that the surface of the as-prepared NiCo_2_O_4_ contains Co^2+^, Co^3 +^, Ni^2 +^, and Ni^3+^. where the atomic ratio of Co to Ni elements is *ca.* 2.2:1, which is close to that in the precursor electrolyte.

**Figure 6:**
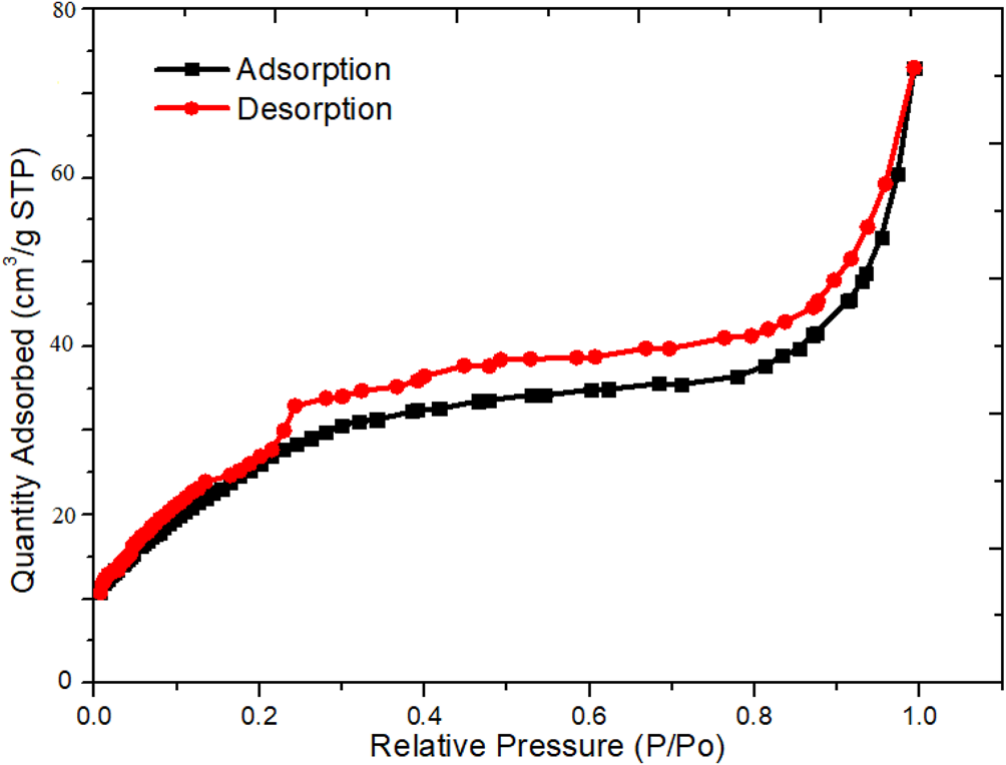
Nitrogen adsorption and desorption isotherms for the NiCo_2_O_4_ sheets.

Nitrogen adsorption/desorption measurements were utilised to examine the porosity and BET surface area and of samples. All the N_2_ adsorption/desorption isotherms in Fig. 6 exhibit a typical IV isotherm with a hysteresis loop in the P/P_0_ range of 0.25–1.0, suggesting the materials have a mesoporous structure. These curves were based on the IUPAC classification of type IV isotherms with loop hysteresis. The specific surface area calculated for the nanocomposite created in the current density of -6.0 mA/cm^2^ were 40.2 m^2^/g. The resulting structure had several advantages in electrochemical supercapacitors. Interconnected NiCo_2_O_4_ sheets grown directly on the nickel foam using electrochemical deposition method provided an integrated and orderly electrode that facilitated the transport of ion and electrons, thereby reducing the electrode resistance.

**Figure 7:**
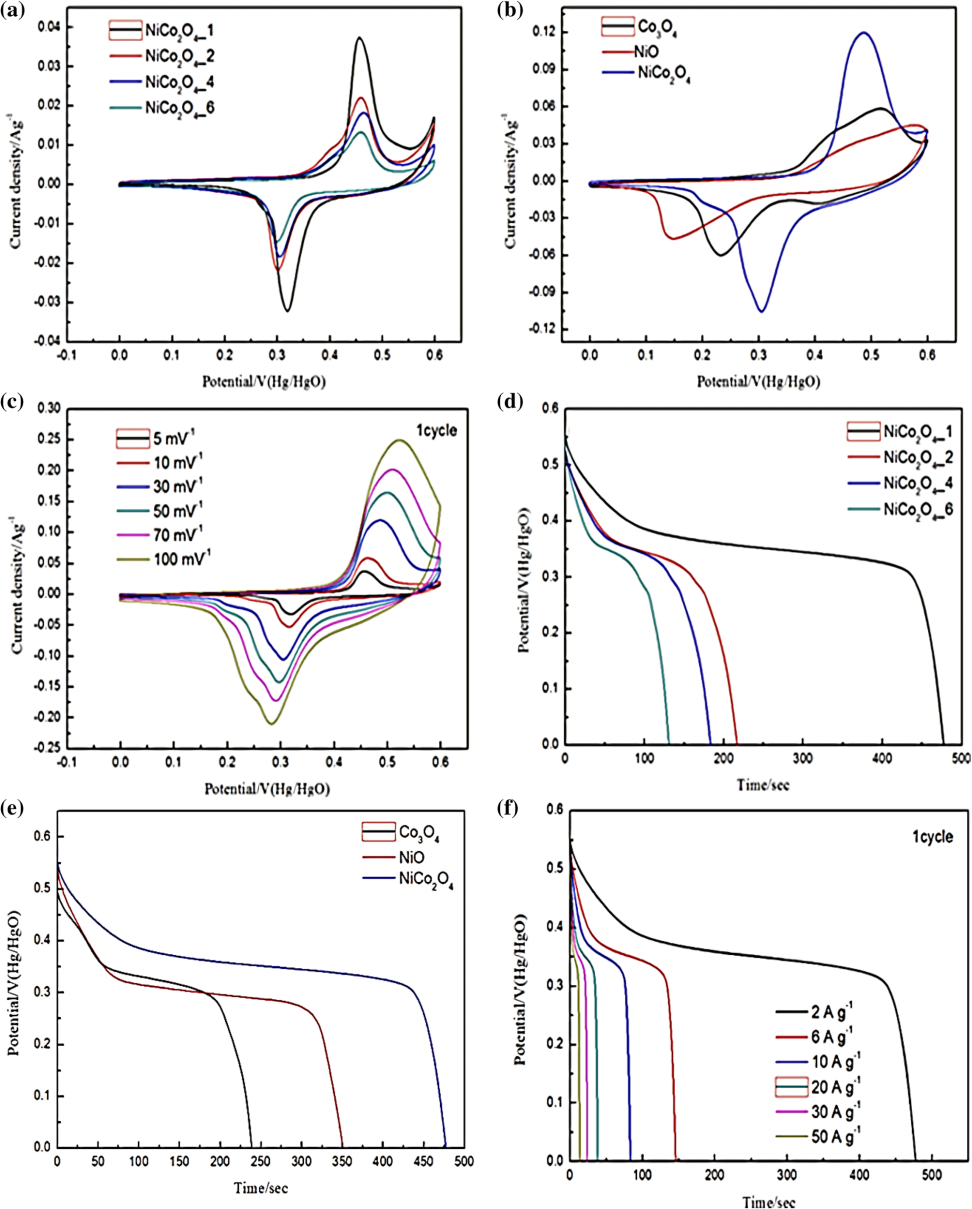
CV curves of (**a**) NiCo_2_O_4_ with different numbers of electrodeposition cycles at 5 mV s^−1^; (**b**) NiCo_2_O_4_, Co_3_O_4_, and NiO as the electrode material at 30 mV s^−1^; and (**c**) NiCo_2_O_4_ at different scan rates. GCD curves of (**d**) NiCo_2_O_4_ with different numbers of electrodeposition cycles at 2 A g^−1^; (**e**) NiCo_2_O_4_, Co_3_O_4_, and NiO as the electrode material at 2 A g^−1^, and (**f**) NiCo_2_O_4_ at different CDs.

Figure 7(a) shows the cyclic voltammetry (CV) curves of NiCo_2_O_4_ with different numbers of electrodeposition cycles at 5 mV s^−1^. The NiCo_2_O_4__1 electrode demonstrates higher peak currents and larger integrated areas compared with the NiCo_2_O_4__2, NiCo_2_O_4__4, and NiCo_2_O_4__6 electrodes. Further, the properties of NiCo_2_O_4__1 shown in Fig. 7(b) indicate better electrochemical capacity than those of Co_3_O_4_ and NiO. Figure 7(c) shows the CV curves of the NiCo_2_O_4_ electrode at scan rates from 5 to 100 mV s^−1^ with a 0–0.6 V potential window (vs. HgCl reference electrode). Even at a relatively high scan rate, a couple of redox peaks are evident, a battery characteristic signifying quick kinetics^16^. The current response enhanced with increasing sweep rate without noticeable alteration in the trend of the CV curves. Furthermore, these peaks are attributed primarily to the faradaic redox reactions involving M–O–OH/M–O (where M signifies Co or Ni)^36^. Figure 7(d, e) presents the galvanostatic charge–discharge (GCD) measurements of Co_3_O_4_, NiO, and NiCo_2_O_4__1 with different numbers of electrodeposition cycles at 2 A g^−1^ from 0 to 0.55 V. Among the samples, NiCo_2_O_4__1 had the best charge–discharge properties. The GCD curves at various CDs are demonstrated in Fig. 7 (f). These CD curves are nonlinear, indicating typical battery-type capacitive behaviour^37,38^. These results confirm that the NiCo_2_O_4__1 electrode had a much higher specific capacitance than the NiCo_2_O_4_ electrodes with more deposition cycles. The remarkable electrochemical performance of the NiCo_2_O_4__1 electrode could be attributed to the excellent adhesion to the nickel foam substrate, with a large surface area and electrical connection of the active material to the current collector to ensure effective accessibility of the electrolyte ions and electrons.

Equation 1 defines a numerical calculation of specific capacitance during current density characterization^39^:

1 $$ C = \frac{I\Delta t}{{m\Delta V}} $$

where *m, I,* Δ*V*, Δ*t,* and C are the active materials mass, discharge current, drop in potential, total discharge time, and specific capacitance, respectively.

**Figure 8:**
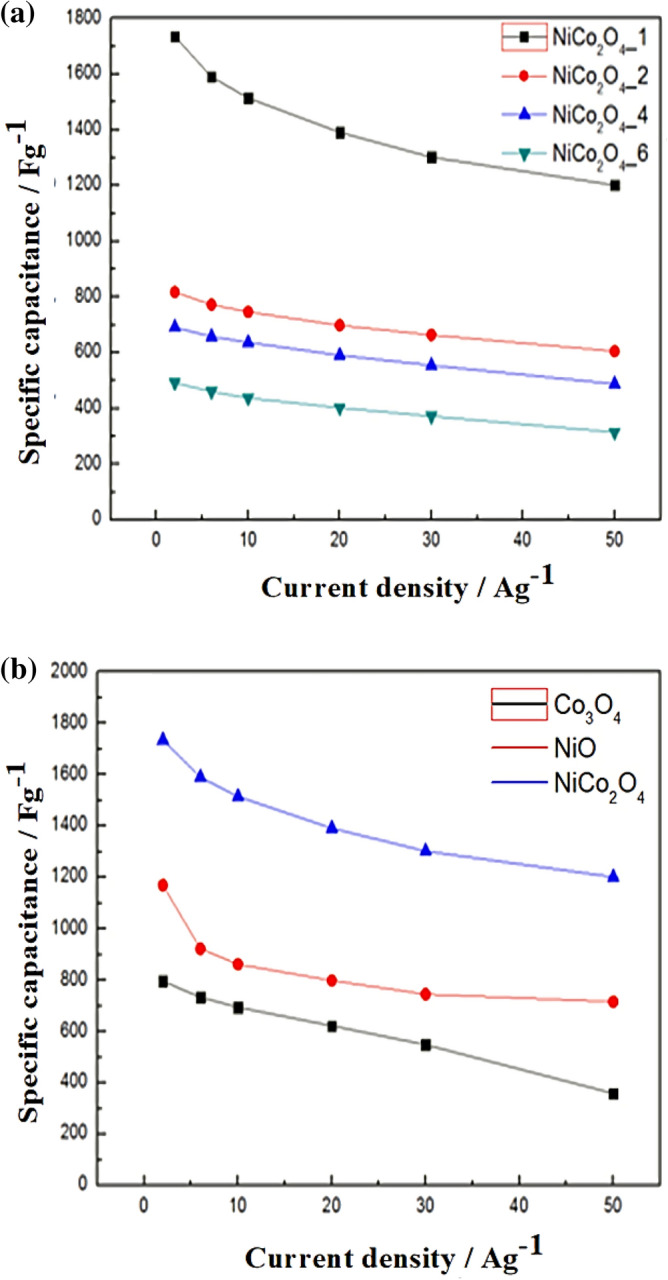
(**a**) Specific capacitance of NiCo_2_O_4_ with different numbers of electrodeposition cycles at different CDs. (**b**) Specific capacitance of NiCo_2_O_4-1_, NiO, and Co_3_O_4_ at different CDs.

Figure 8 (a) shows the specific capacitance of NiCo_2_O_4_ with different numbers of electrodeposition cycles at different current densities. NiCo_2_O_4__1 shows a superior specific capacitance. Figure 8 (b) shows the specific capacitance of NiCo_2_O_4__1, NiO, and Co_3_O_4_, and the NiCo_2_O_4__1 nanosheet electrode shows excellent capacitance values of 1734.9, 1590.5, 1514.7, 1391.3, 1302.4, and 1201.8 F g^−1^ at CDs of 2, 6, 10, 20, 30, and 50 A g^−1^, respectively. This shows that when the charge–discharge rate increases from 2 to 50 A g^−1^, around 87.3% of the capacitance is retained.

**Figure 9:**
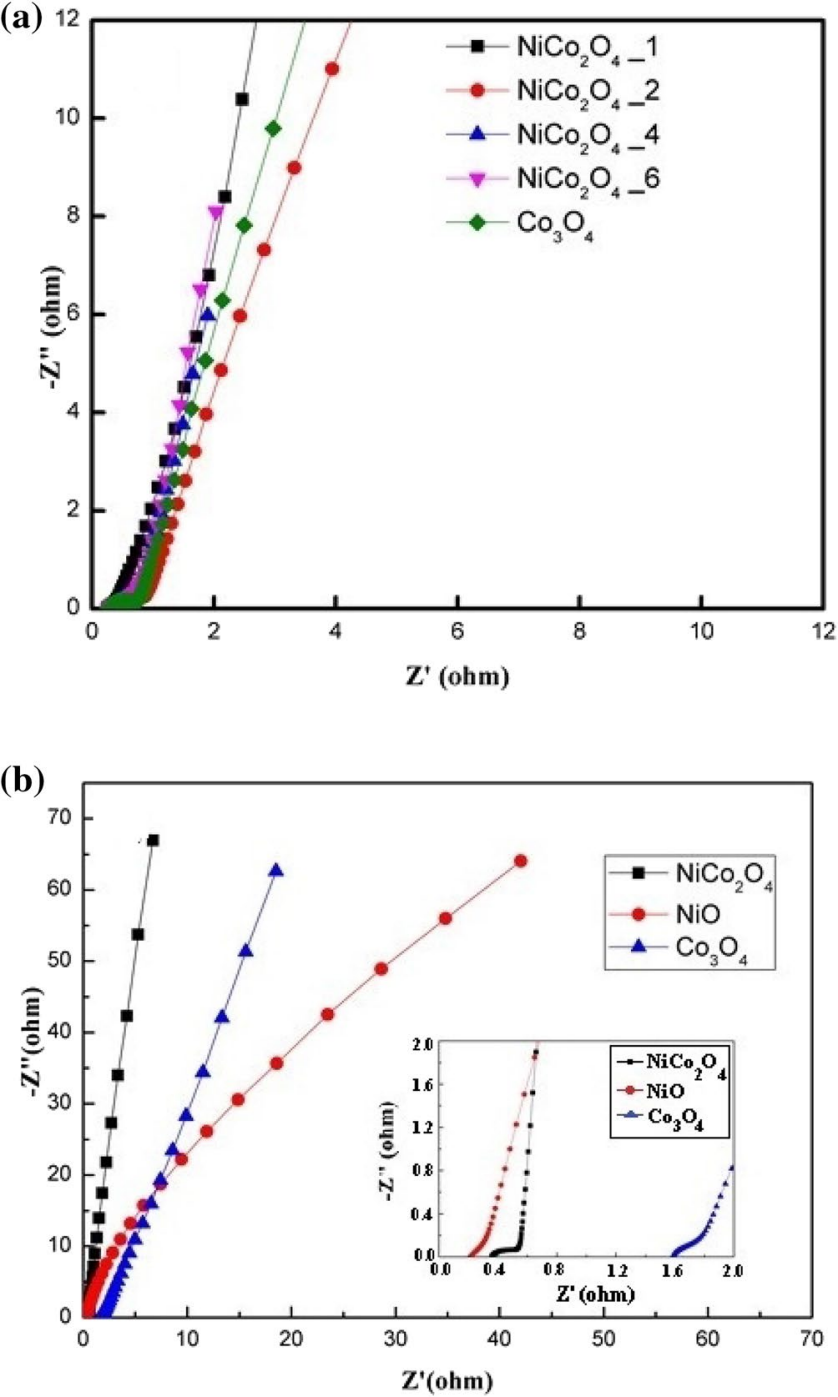
Nyquist impedance plots of (**a**) NiCo_2_O_4_ with different numbers of deposition cycles and (**b**) NiCo_2_O_4_, NiO, and Co_3_O_4_.

Electrochemical impedance spectroscopy (EIS) tests were carried out to examine the ion transport properties of the synthesised materials in the 0.01 Hz–100 kHz frequency range. Figure 9 shows the EIS results for the NiCo_2_O_4__*n* (*n* = 1, 2, 4, and 6), NiO, and Co_3_O_4_ materials. The Nyquist plots of the electrodes contain straight and semicircular curves in the low and high frequency regions, respectively^40–43^. The intercept of *Z*_0_ (the real axis) with the semicircle, in the high-frequency region, is identical to *R*_s_ (the internal resistance), which comprises of the ohmic resistance of the active materials, the resistance of the electrolyte, and the contact resistance at the interface of the active material/nickel foam. The internal resistance values of the NiCo_2_O_4__1, NiO, and Co_3_O_4_ electrodes were 0.35, 0.22, and 1.58 Ω, respectively. The semicircle represents *C*_dl_ (the double-layer capacitance), which is related to the surface properties of the electrode, and the semicircle diameter indicates *R*_ct_ (the charge transfer resistance), which is related to the corresponding faradaic reactions at the interface of the electrode–electrolyte. On the contrary, the slope of the curves in the low frequency region signifies the Warburg resistance, which is related to the diffusion of the electrolyte in the electrodes. It is known that the electrochemical performance of supercapacitors can be effectively enhanced by reducing this resistance. Based on the results of GCD, an extraordinary specific capacitance of 1734.5 F g^−1^ was obtained at a current density of 2 A g^−1^. The CV curves of NiCo_2_O_4_ with different numbers of electrodeposition cycles at 5 mV s^−1^ are given in Fig. 9(a). The NiCo_2_O_4__1 electrode demonstrates higher peak currents and larger integrated areas compared with the NiCo_2_O_4__2, NiCo_2_O_4__4, and NiCo_2_O_4__6 electrodes. The internal resistance of the NiCo_2_O_4__1 electrode was only 0.35 Ω. These results indicate that NiCo_2_O_4__1 electrode has good electrochemical performance.

**Figure 10:**
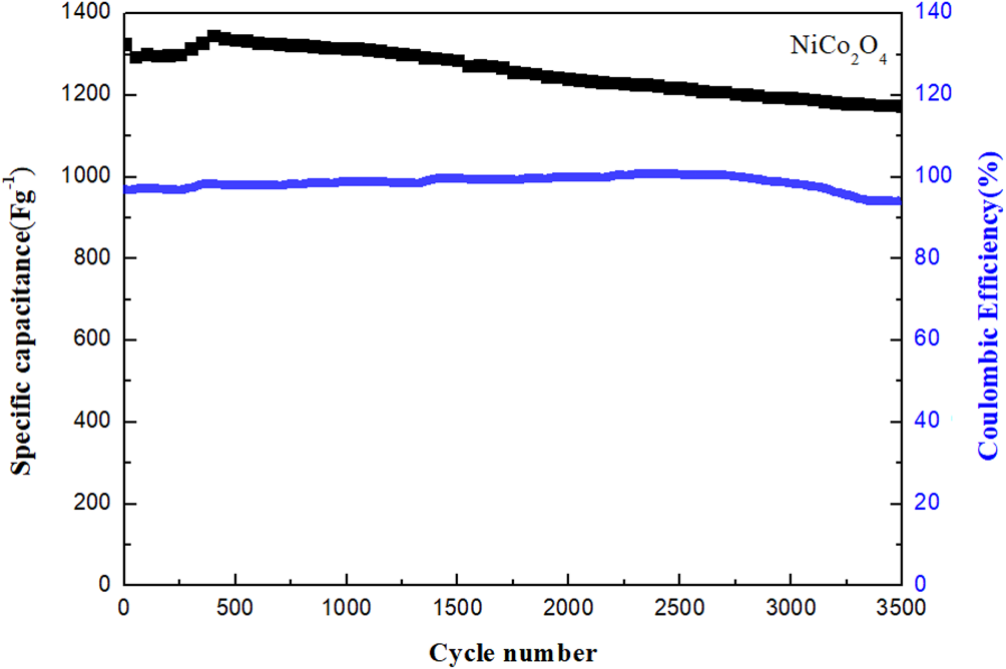
Cycling stability and Coulombic efficiency of the NiCo_2_O_4_ electrode at a CD of 30 A g^−1^ for 3500 cycles.

The cycle life performance of NiCo_2_O_4_ at a CD of 30 A g^−1^ for 3500 cycles is presented in Fig. 10. The NiCo_2_O_4_ deposited on the nickel foam electrode shows steady cycling stability. From the calculation for the discharge curves, an approximate decrease of only 12.7% in the specific capacitance value after 3500 cycles is obtained. At low current densities, some side reactions occurred during the electrochemical redox reaction, leading to incomplete discharge. As the current density increased, the charge and discharge time decreased. The electrochemical process was mainly affected by the electric double layer, so the Coulomb efficiency increased as well^9^. Given the superior electrochemical behaviour of the NiCo_2_O_4_ electrode, it is the best choice for use in electrochemical supercapacitors characterised by both excellent rate capability and long cycle life.

**Figure 11:**
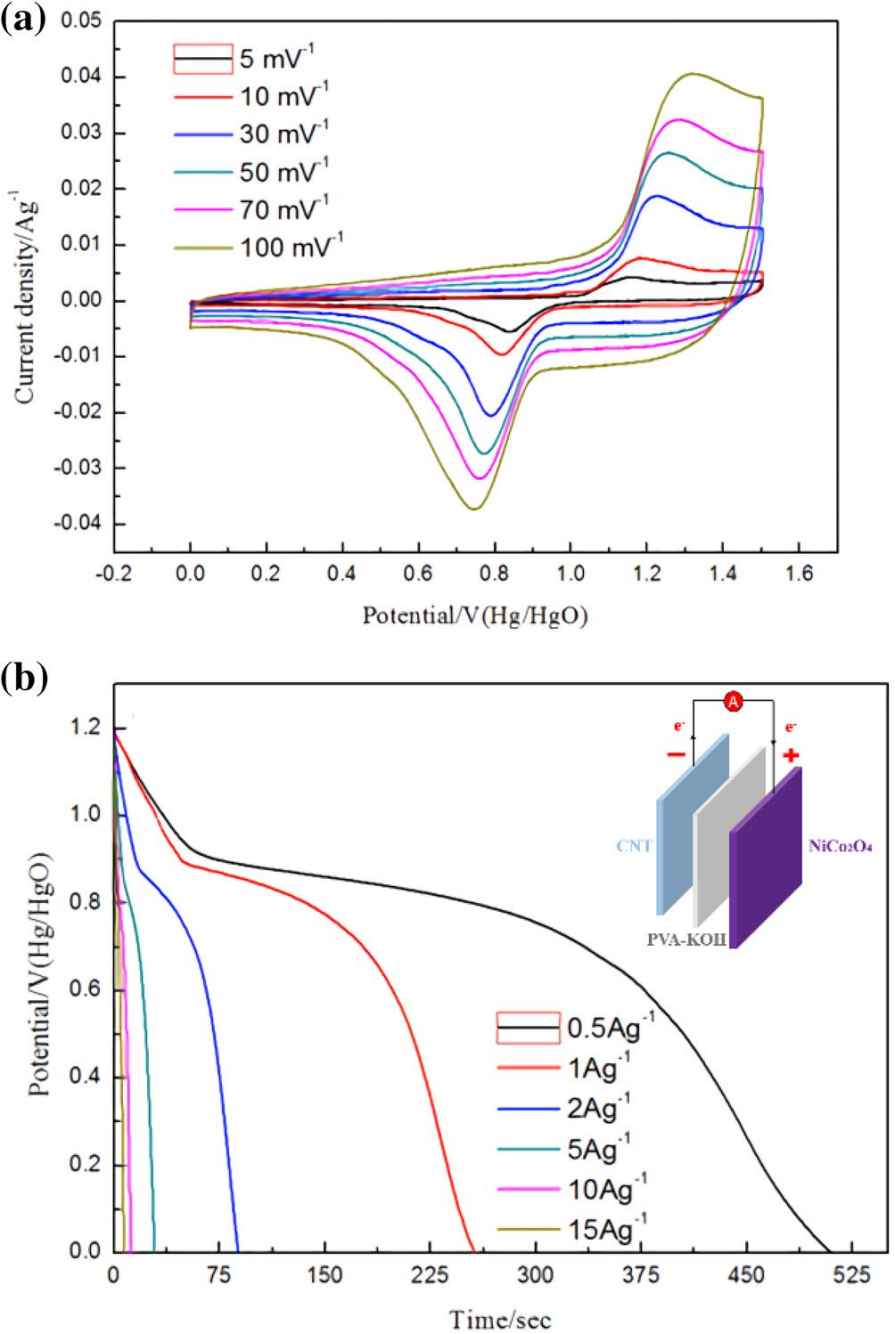
(**a**) CV curves of the ASC device. (**b**) Charge–discharge curves of the ASC device. The embedded picture is a schematic of the ASC device.

**Figure 12:**
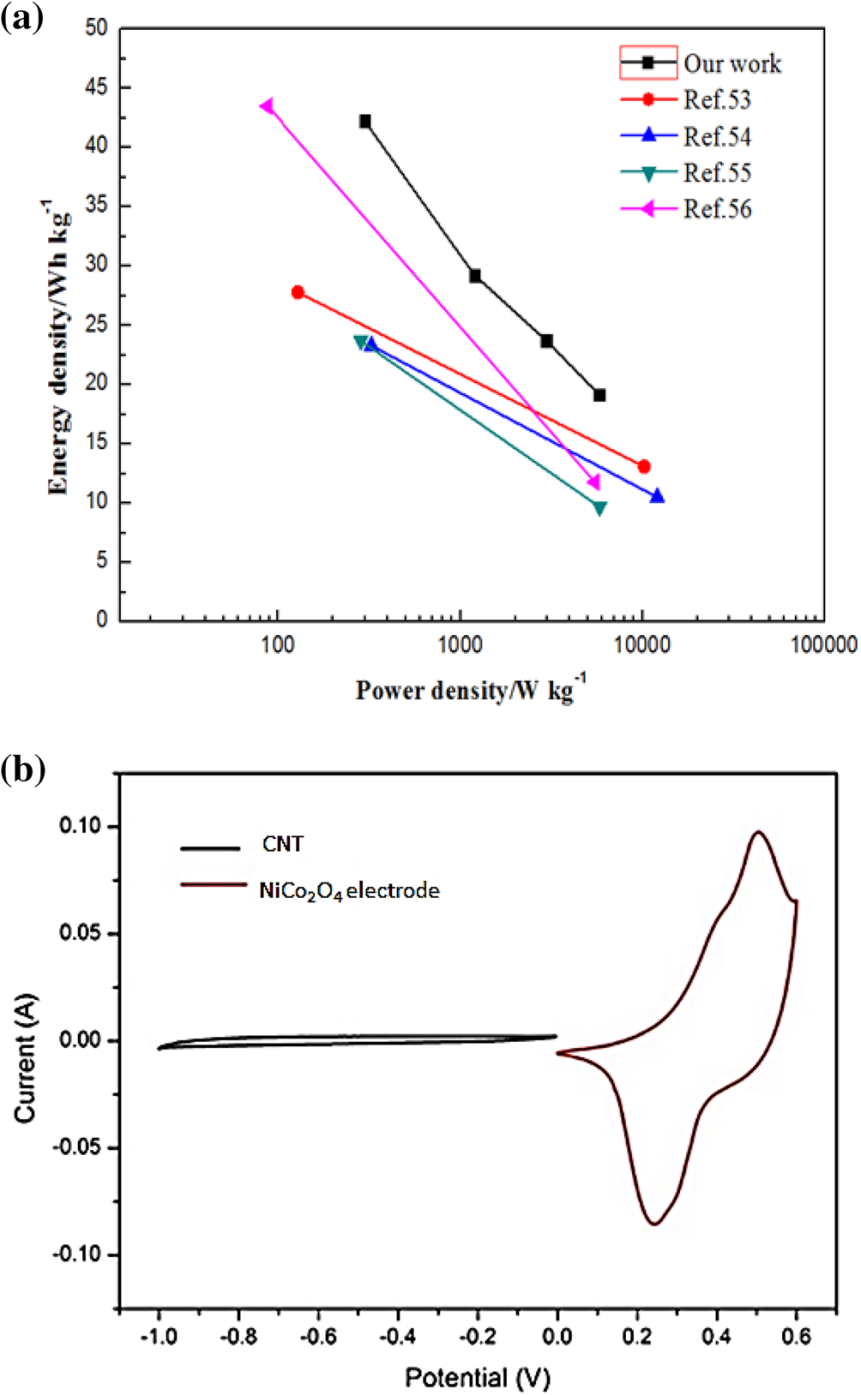
(**a**) Power and energy density plot (Ragone plot) of the current ASC device at different charge–discharge rates compared with the literature. (**b**) CV curves of the CNT and NiCo_2_O_4_ electrodes in a three-electrode system at a scan rate of 5 mV s^−1^.

Furthermore, to study the practical performance of the synthesised electrode in this work, asymmetric supercapacitors (ASCs) were assembled, in which carbon nanotubes (CNTs) and NiCo_2_O_4_ were the cathode and anode, respectively, with a polyvinyl alcohol-KOH gel polymer electrolyte. Figure 11(a) displays the CV curves of the anode and cathode at 5–100 mV s^−1^ scan rates. Figure 12(b) indicates that the NiCo_2_O_4_ and CNT electrodes operate in voltage ranges of 0.0–0.6 V and − 1.0 to 0.0 V, respectively. Consequently, the NiCo_2_O_4_/CNT ASC can operate in a 1.6 V voltage range^12^. As the scan rate increases from 5 to 100 mV s^−1^, the shape of the CV curve does not change, indicating that the device has good and rapid charge–discharge properties. The charge–discharge behaviour of the device is depicted in Fig. 11(b). These charge–discharge curves are nonlinear, indicating battery-type capacitive behaviour. Equations 2 and 3 were used to further investigate the excellent rate capability and high capacitance to evaluate the performance indicators of specific power density (*P*) and specific energy density (*E*) from the discharge curves^13,44–47^.

2 $$ E = {1/2}\left( {C \times \, \Delta V^{{2}} } \right) $$

3 $$ P = { 36}00 \, (E/\Delta t) $$

where Δ*V, C,* and Δ*t* are the potential drop during discharge, specific capacitance derived from the charge–discharge calculations, and full discharge duration, respectively.

Figure 12 depicts the power and energy density plot (Ragone plot) of the devices at different charge–discharge rates. The calculated energy densities of the NiCo_2_O_4_/CNT ASC were 42.25, 41.43, 29.25, 23.73, and 19.16 W h kg^−^1 at power densities of 298.79, 596.66, 1196.52, 2966.29, and 5796.04 W kg^−1^, respectively. These findings indicate that the ASC device attained a greater energy density than reported devices, such as colloidal quantum dots/NiCo_2_O_4_/activated carbon (AC) (27.8 W h kg^−1^ at 128 W kg^−1^)^48,49^, NiCo_2_O_4_-reduced graphene oxide/AC (23.32 W h kg^−1^ at 324.9 kW kg^−1^)^50^, Ni_x_Co_1−x_ LDH–zinc tin oxide/AC (23.7 W h kg^−1^ at 284.2 W kg^−1^)^51^, and CoO@polypyrrole/AC (43.5 W h kg^−1^ at 87.5 W kg^−1^)^52^.

## Conclusions

In summary, NiCo_2_O_4_ was synthesised on nickel foam via electrodeposition. NiCo_2_O_4_ was found to have superior specific capacitances of 1734.9 and 1201.8 F g^−1^ at CDs of 2 and 50 A g^−1^, respectively, with great cycling stability (only 12.7% loss after 3500 cycles). Furthermore, a high performance solid-state ASC was built utilising NiCo_2_O_4_ and CNTs as the anode and cathode, respectively, and the solid-state polyvinyl alcohol–KOH gel as an electrolyte. A specific capacitance of 212.47 F g^−1^ was attained at a 0.5 A g^−1^ CD. Moreover, the ASC exhibited a high energy density (42.25 W h kg^−1^) at a 298.79 W kg^−1^ power density and a high power density (5,796.04 W kg^−1^) at a 19.16 W h kg^−1^ energy density.

## Materials and methods

### Preparation of nickel cobaltite

The electrochemical deposition was carried out at ambient temperature to synthesize NiCo_2_O_4_ nanosheets onto nickel foam in a 3-electrode cell in which nickel foam, saturated calomel, and Pt foil utilized as working, reference, and counter electrodes, respectively. Nickel–Cobalt layered double hydroxide precursor was deposited over nickel foam in an aqueous mixed electrolyte of 2 mM cobalt nitrate and 1 mM nickel nitrate using a ZIVE SP2 electrochemical workstation.

Versatile numbers of cycles like 1, 2, 4, and 6 cycles were selected for electrodeposition of working electrodes with the potential of − 1.2–0.5 V (vs. SCE). The nickel foams were ultrasonically cleaned and rinsed three times with distilled water and ethanol after electrodeposition and then dried at ambient temperature. Then, the electrodeposited working electrodes was placed in a muffle furnace and annealed for two hours at 300 °C, to transform the coating into interconnected mesoporous NiCo_2_O_4_ nanosheets; it was carefully weighed after annealing (Fig. 1). The obtained NiCo_2_O_4_ specimens are called as NiCo_2_O_4__*n* (where *n* is the number of cycles, *n* = 1, 2, 4, and 6), and henceforth “NiCo_2_O_4_” denotes the *n* = 1 sample when NiCo_2_O_4_ is compared with other materials.

### Preparation of flexible solid electrolyte

The solution-casting technique was used to prepare the potassium hydroxide and PVA polymer electrolyte. 2 g of polyvinyl alcohol was added into 30 ml of double-distilled water and stirred for three hours at 80 °C. Then 6 M potassium hydroxide was mixed the prepared solution with agitation at room temperature for 3 h. After dissolution was complete, the final mixture was constantly agitated until a homogeneous viscous product was obtained. Finally, to attain a jelly electrolyte, the mixture was transferred to an oven (vacuum) and kept at 70 °C overnight.

### Characterization of NiCo_2_O_4_

The morphology and microstructure of specimens were examined by FESEM (FESEM, LEO-1550) equipped with EDS at a 5 kV applied voltage. XRD tests were carried out (Bruker D8 Advance X-ray diffractometer) with Cu K-α radiation (λ = 0.154056 nm) at 30 mA and 40 kV. The speed of scanning was 5° min^−1^ with 0.02° steps. Elemental mapping (FEI Talos microscope operating at a 200 kV accelerating voltage), high-angle annular dark-field scanning, and TEM (HAADF-STEM-EDS) were used to characterize the specimens. Thermo VG Escalab 250 photoelectron spectrometer was used for XPS analysis. The pore structures were evaluated by N^2^ adsorption at 77 Kusing volumetric equipment (Quantachrome AS-1-MP) after pre-evacuation for 2 h at 423 K while maintaining a base pressure of 10^–4^ Pa.

### Electrochemical characterizations

The electrochemical characterizations containing electrochemical impedance spectroscopy (EIS), galvanostatic charge–discharge (GCD), and cyclic voltammetry (CV) were carried out in a conventional three-electrode configuration at ambient temperature in which the NiCo_2_O_4_ on Ni foam electrode was utilized as the working electrode, and a Hg/HgO and Pt foil were the reference and counter electrodes. The electrochemical characterizations were done using a 6 M potassium hydroxide solution with the aid of ZIVE SP2 workstation (10 μHz–4 MHz). The voltage range was from 0 to 0.6 V vs. Hg/HgO for the NiCo_2_O_4_ electrode. To examine the energy storage performance of the working electrode for practical application, the anode electrode material was nickel cobalt oxide, and the cathode electrode material was carbon nanotubes (CNTs). The voltage range was from 0 to 1.5 V vs. Hg/HgO for the CNT electrode.
